# The impact of the estrous cycle on the vaginal microbiota of dairy cows

**DOI:** 10.1371/journal.pone.0359551

**Published:** 2026-10-05

**Authors:** Anne Kemmer Souza, Adèle Giroux, Amanda Fonseca Zangirolamo, Ricardo Guella Droher, Francieli Gesleine Capote Bonato, Amauri Alfieri, Gustavo Zamberlam, Marcelo Marcondes Seneda, Márcio Costa

**Affiliations:** 1 National Institute of Science and Technology for Dairy Production Chain (INCT–LEITE), Universidade Estadual de Londrina, Londrina, Paraná, Brazil; 2 Laboratório de Biotecnologia da Reprodução Animal (REPROA), Departamento de Clínicas Veterinárias (DCV), Centro de Ciências Agrárias (CCA), Universidade Estadual de Londrina (UEL), Londrina, Paraná, Brazil; 3 Département de biomédecine vétérinaire, Faculté de médecine vétérinaire (FMV), Université de Montréal, Saint-Hyacinthe, Quebec, Canada; Sathyabama Institute of Science and Technology, INDIA

## Abstract

The present study investigated the vaginal microbiota of dairy cows throughout the estrous cycle and early gestation, under natural physiological conditions and without hormonal manipulation. Vaginal swabs were collected at estrus (D0), metestrus (D3), diestrus (D15), and Day 19 post-insemination (representing proestrus for non-pregnant cows or early gestation for pregnant cows). Pregnancy diagnosis was performed on day 31 post-insemination. Microbial characterization was performed using 16S rRNA gene sequencing, followed by analyses of alpha- and beta-diversity, community composition, and differential abundance. Overall, the vaginal microbiota exhibited high inter-individual variability and only subtle shifts across reproductive phases. Beta diversity analysis revealed modest but significant compositional rearrangements between estrus vs. proestrus (strictly within the non-pregnant cohort) and estrus vs. diestrus. Differential abundance testing identified two taxa enriched during pregnancy (*Muribaculum* and *Hoministercoradaptatus*), indicating potential microbial biomarkers associated with the gestational environment. These findings demonstrate that, in naturally cycling cows, physiological hormonal oscillations induce only mild microbial changes, whereas individual factors appear to play a dominant role in shaping the vaginal community. The work contributes to defining baseline microbial patterns in non-manipulated animals and highlights the importance of distinguishing natural variation from hormonally induced effects in reproductive microbiome research.

## Introduction

The vaginal microbiota in female cattle represents an essential component of reproductive health, acting as a protective barrier against pathogens and modulating local immunological interactions [1]. Historically, much of the research on microbiota has focused on detecting pathogenic bacteria, overlooking the role of beneficial microbial communities. However, as understanding deepens, it becomes evident that not all bacterial presence is harmful; rather, many communities can serve as positive biological markers of health and reproductive well-being.

In high-producing dairy cows, microbial composition can vary throughout the estrous cycle, in response to natural hormonal changes that affect pH, vaginal secretion profile, and local immunity. These physiological oscillations can modulate bacterial composition and diversity, resulting in specific microbial profiles for each phase of the cycle [2]. However, the use of hormonal protocols for estrus synchronization, a practice widely adopted in intensive production systems, can significantly interfere with this natural dynamic [3]. Interventions with progesterone, estrogens, prostaglandins, and gonadotropins alter the vaginal environment by modifying mucus, blood flow, and the immune response, potentially producing artificial changes in microbial composition. These alterations can mask or attenuate spontaneous variations in the microbiota, making it difficult to understand its natural behavior and its real influence on fertility [4,5].

From this perspective, studies show that both estradiol (E2) and progesterone (P4) can directly modify the bovine vaginal environment, altering mucous secretions, epithelial permeability, and local immune dynamics, thereby producing artificial patterns during reproductive protocols [6]. Subtle changes in bacterial abundances have been associated with estradiol use [7], and progesterone interventions, particularly via intravaginal devices, have been shown to selectively modify certain bacterial taxa [3]. In addition, endogenous variations in estrogen and progesterone have been associated with relevant uterine changes in beef cattle [8].

Therefore, investigations that characterize the vaginal microbiota of high-producing dairy cows without exogenous hormonal interference are essential. By analyzing the estrous cycle phases (e.g., follicular and luteal) in a naturalistic manner, it is possible to delineate the physiological fluctuations in bacterial communities and identify endogenous biomarkers of reproductive health. This knowledge is particularly relevant for understanding host–microbiome interactions within the female reproductive tract and for identifying microbial signatures associated with fertility and pregnancy establishment [1,9–11].

The objective of this study was to investigate the vaginal microbiota of high-producing dairy cows under natural physiological conditions without exogenous hormonal interference. This information contributes to a more precise understanding of the relationship between the microbiome and the bovine reproductive tract.

## Materials and methods

### Ethics statement

The study was approved by the Animal Ethics Committee of the State University of Londrina (CEUA/UEL), Brazil (Protocol No. 060.2024; approval date: December 4, 2024). All animal procedures were conducted in accordance with Brazilian legislation and the guidelines established by the National Council for the Control of Animal Experimentation (CONCEA). Prior to the initiation of the study, written authorization was obtained from the owner of the farm and the animals, who was informed about the objectives of the research, the study procedures, and their potential implications before voluntarily consenting to the inclusion of the animals in the project. The signed Statement of Acknowledgment and Authorization is provided as S2 File. No deviations from the approved protocol occurred during the study. Additional information regarding the ethical, cultural, and scientific considerations specific to inclusivity in global research is included in the Supporting Information (S1 File).

### Animals

For the experiment, 21 Holstein cows, aged 4–8 years and weighing 450–650 kg, from a dairy farm in Castro, Paraná, Brazil, were used. The selected cows were multiparous and high milk producers. According to the farm health records, the cows had no documented recent history of reproductive disease and showed no clinical signs of reproductive disorders at the time of enrollment. In addition, none of the animals had received antibiotic treatment during the period immediately preceding sample collection. Regarding management, the cows were milked twice daily and maintained in a semi-confinement system. The animals spent part of the day in paddocks with ryegrass (*Lolium multiflorum*) pasture, and after each milking, they received a diet offered in a trough consisting of commercial concentrate (16% crude protein) and corn silage.

### Reproductive management

As an essential part of this work, the females were not subjected to hormonal protocols. Estrus detection was performed using a monitoring collar (Allflex, Livestock Intelligence TM). This system consists of equipment attached to a collar worn on the animal's neck that records activity, rumination, and panting, and transmits the data to management software. This data is used to detect estrus and establish the optimal time for insemination. On the day of estrus, the cows were inseminated in accordance with the farm's reproductive management protocol. Pregnancy diagnosis was performed thirty days after insemination by transrectal ultrasonography. The cows remained with the rest of the herd throughout the experiment, and no changes were made to the reproductive, sanitary, feeding, and zootechnical management already in place on the farm.

### Sample collection

Vaginal bacterial sampling of each cow was performed using a sterile swab, which was introduced into the female's vagina after cleaning the perineal region with a disposable paper towel soaked in 70% alcohol. To reduce contamination, the perineal region was cleaned, and using clean gloves, the vulvar lips were opened and the swab was introduced. Fifteen circular movements were performed on the vaginal mucosa, after which the swab tip was placed into a sterile Eppendorf tube. Samples were collected in duplicate and placed in a liquid nitrogen tank for transport to the Animal Reproduction Biotechnology Laboratory (REPROA) at the State University of Londrina, Parana, Brazil.

Ovarian activity was verified by transrectal ultrasound after each sample collection. The first sample was collected on the day of estrus (D0). Subsequent samples were collected on day 3 (D3, metestrus) and day 15 (D15, diestrus) post-insemination for all cows. On day 19 (D19), the collection corresponded to the proestrus phase strictly for cows that did not conceive, whereas for pregnant cows, this timepoint represented early gestation under a sustained luteal phase. Finally, transrectal ultrasonography was performed on day 31 to confirm pregnancy status (Fig 1).

**Fig 1:**
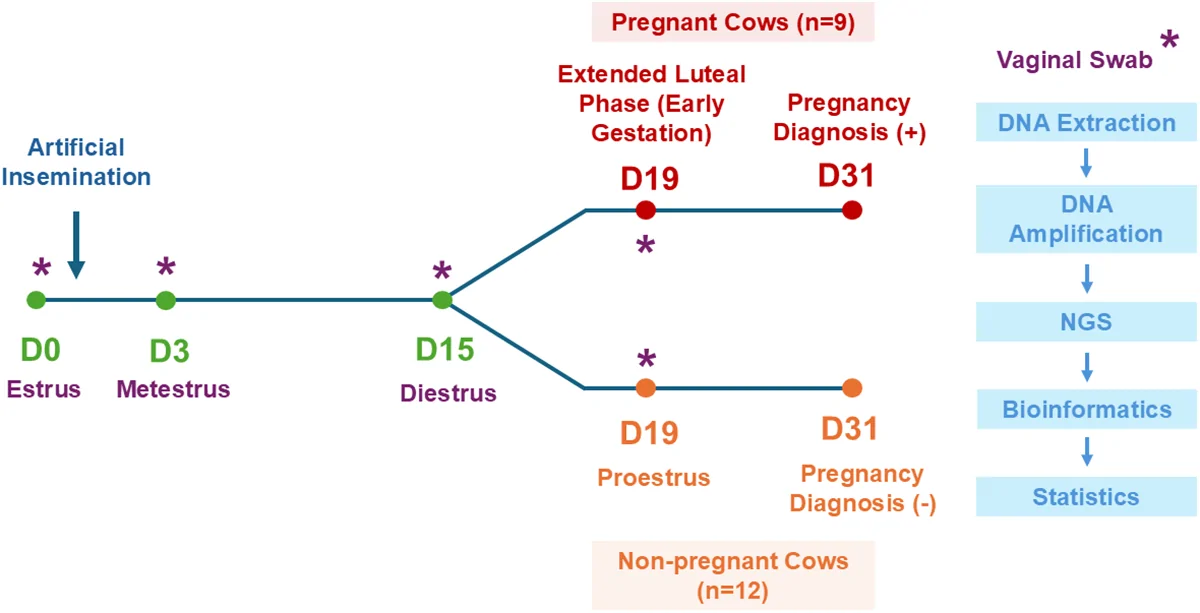
Timeline of the experiment. Illustrating the days of vaginal swab collection and the laboratory processing workflow for microbiota identification. D0 = estrus and artificial insemination; D3 = metestrus; D15 = diestrus; D19 = proestrus; D31 = pregnancy diagnosis by ultrasonography. Note: Day 19 (D19) represents proestrus strictly for non-pregnant cows, while pregnant cows remain in a sustained luteal phase (early gestation).

### Microbiota analysis

DNA extraction was performed using the commercial PowerSoil^®^ kit (QIAGEN). The samples were thawed at room temperature (22 ℃), and the swab was added to the PowerBead tube. Subsequently, 60 μl of solution C1 was added to the tubes and vortexed at maximum speed for 10 minutes. The following steps were according to the manufacturer's instructions. DNA quality was confirmed by spectrophotometry, and 16S rRNA gene amplification was confirmed by electrophoresis on a 1.0% agarose gel. PCR amplification of the V4 region of the 16S rRNA gene was carried out using the primers 515F (GTGCCAGCMGCCGCGGTAA) and 806R (GGACTACHVGGGTWTCTAAT). Sequencing was performed at Genome Quebec using an Illumina MiSeq platform with 250-cycle read ends, with reads fully overlapping.

Bioinformatics analysis was performed using Mothur [12] (version 1.48.3) following the previously described Standard Operating Procedure [13]. Contig assembly was performed excluding sequences longer than 300 bp, containing any base-pair ambiguities, and having polymers longer than 8 bp. The sequences were aligned against the SILVA 16S rRNA reference database (release 138.2), clustered at 97% similarity, and chimeras were removed with the vsearch tool. Reads were then classified using the Ribosomal Database Project (RDP) version 19 (2023).

The number of different taxa (Observed OTUs) and the Shannon index were used to characterize alpha diversity, and the Bray-Curtis index to characterize beta diversity (comparison of taxonomic composition between samples). A two-dimensional principal coordinate analysis (PCoA) plot was generated to visualize sample similarity (beta diversity). The most abundant bacteria (>1%) were visualized using bar charts showing the relative abundance of the main genera in each sample, and taxa with statistically significant differences were visualized in boxplots.

For the statistical analysis, to accurately account for the diverging physiological and endocrine environments post-insemination, samples from cows that became pregnant collected at D3, D15, and D19 were grouped together and classified as PREG (pregnant group). Consequently, the ‘Proestrus’ group strictly comprised the D19 samples from the 12 non-pregnant cows that underwent regular luteolysis and entered the follicular phase.

Alpha-diversity parameters were compared over time using a repeated-measures Kruskal-Wallis test with the Dunn’s test to identify specific pairwise differences between stages, whereas the PERMANOVA test was used to investigate compositional differences (beta diversity). The Linear Models for Differential Abundance (LinDA) [14] was used to compare the relative abundances of taxa associated with a specific stage of the estrous cycle and to identify potential markers of fertility. For that matter, data on pregnancy status were used to classify cows into two categories (pregnant or non-pregnant), to compare microbiota composition on Day 0 (artificial insemination).

Supplementary Tables. Abundance table, taxonomic classification, and sample metadata. The supplementary dataset contains three complementary files: (S3 File) an OTU abundance table showing the number of reads of each operational taxonomic unit (OTU) across samples; (S4 File) a taxonomy file providing the taxonomic classification assigned to each OTU, from phylum to genus; and (S5 File) a metadata file containing characteristics associated with each sample.

## Results

DNA sequencing yielded 5,441,617 raw reads from 84 vaginal samples. After bioinformatics processing and quality filtering, 4,074,819 high-quality sequences were retained for downstream analyses. Sample normalization was then performed by subsampling to 10,326 sequences per sample, corresponding to the lowest sequencing depth observed among the samples. Following artificial insemination at D0, pregnancy diagnosis confirmed that 9 cows became pregnant, whereas the remaining animals were classified as non-pregnant.

### Relative abundances

Overall, high individual variability was observed, indicating that each cow exhibited a distinct vaginal microbial profile regardless of reproductive phase (Fig 2). There was also substantial within-cow variability, as shown in Fig 3.

**Fig 2:**
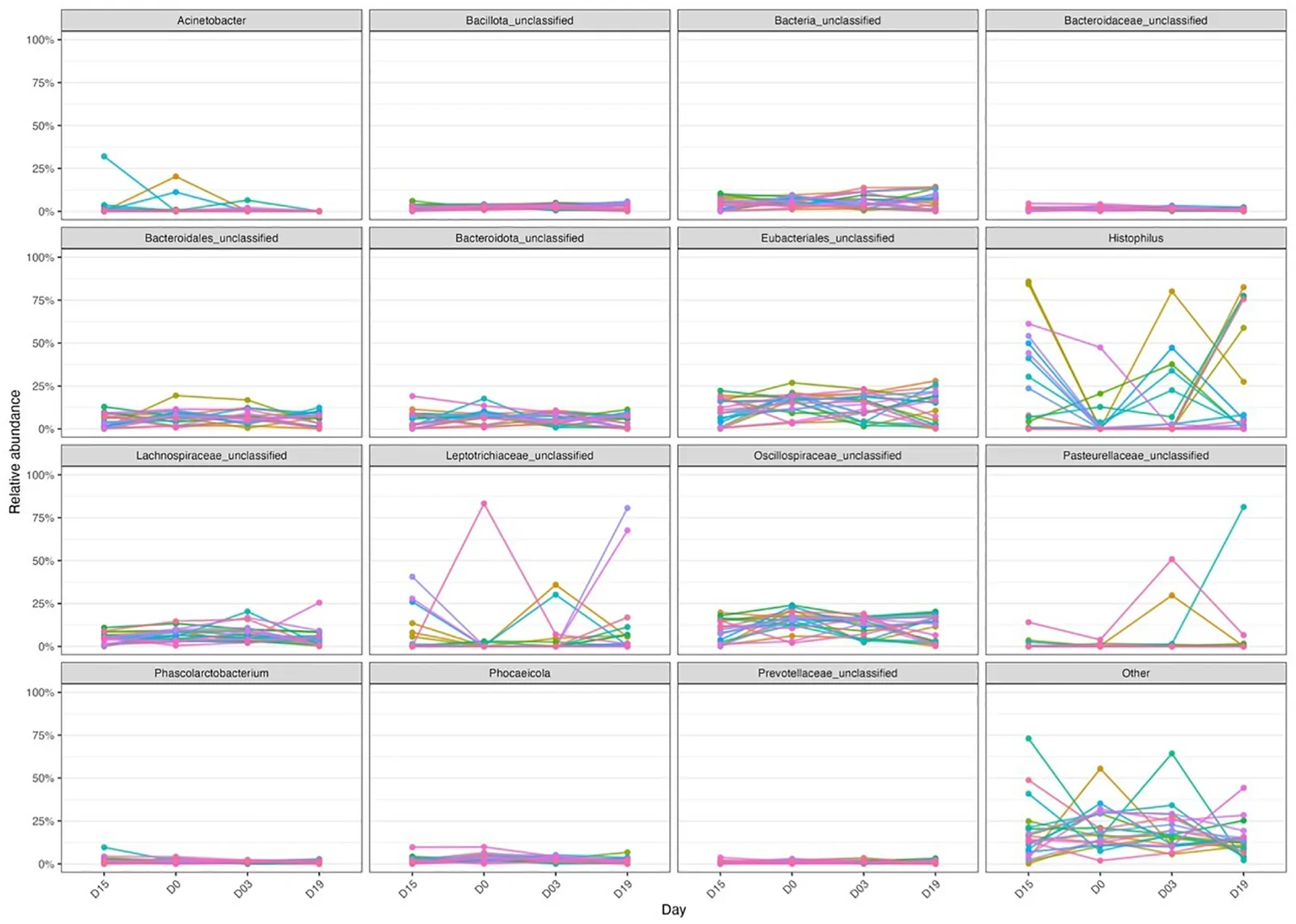
Longitudinal dynamics of the 15 most abundant taxa present in the vaginal microbiota of dairy cows. Relative abundance of the 15 most abundant bacterial taxa identified in vaginal samples collected throughout the reproductive cycle. D0 = estrus and artificial insemination; D3 = metestrus; D15 = diestrus; D19 = proestrus, strictly for non-pregnant cows, while pregnant cows remain in a sustained luteal phase (early gestation). Each bar represents one sample, and colors indicate different bacterial taxa.

**Fig 3:**
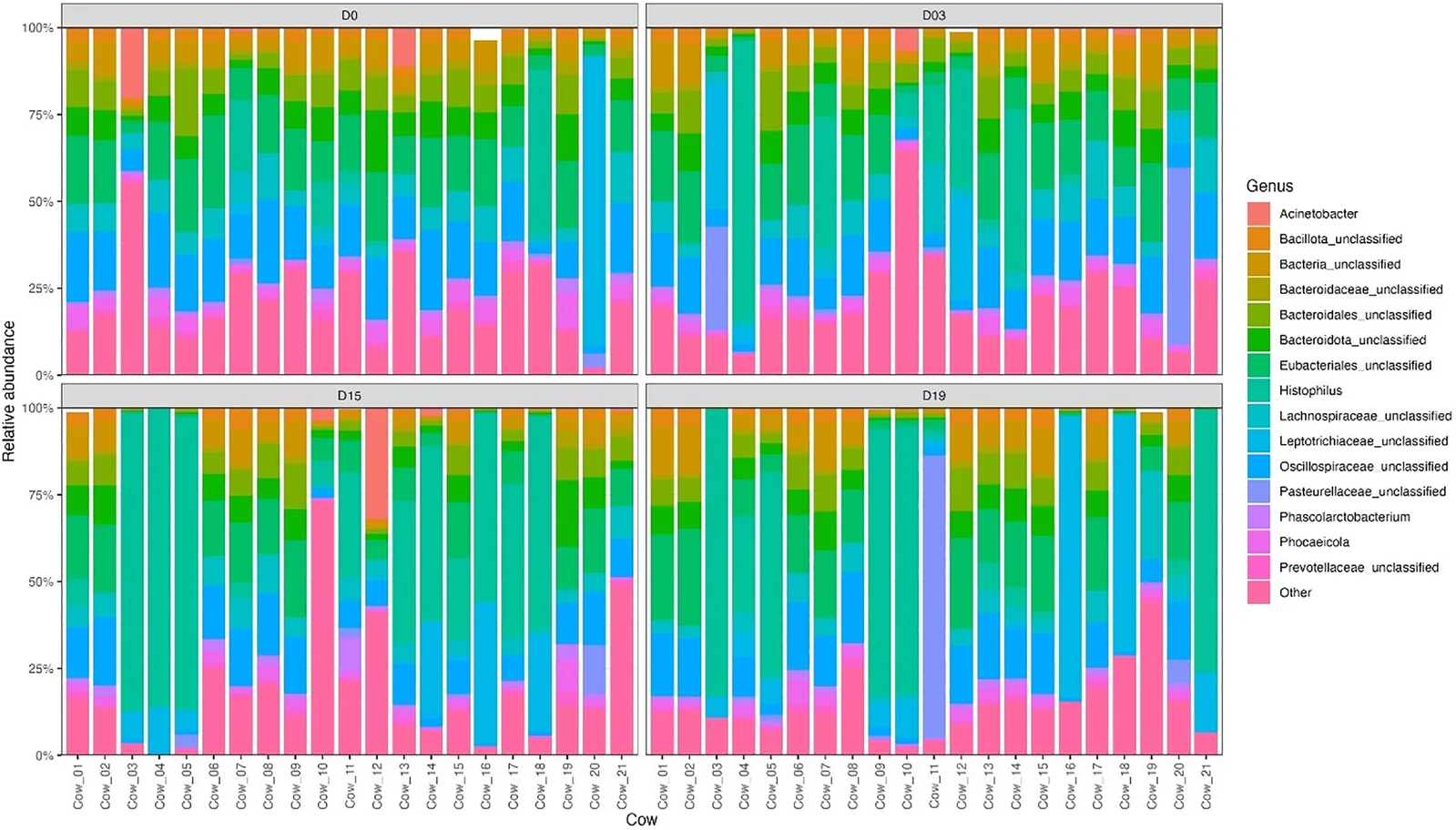
Individual relative abundances of the 15 most abundant taxa present in the vaginal microbiota of dairy cows. Relative abundance of the 15 most abundant bacterial taxa identified in vaginal samples collected throughout the reproductive cycle. D0 = estrus and artificial insemination; D3 = metestrus; D15 = diestrus; D19 = proestrus, strictly for non-pregnant cows, while pregnant cows remain in a sustained luteal phase (early gestation). Each panel represents an individual animal.

For the differential detection analysis (Linda), samples from pregnant cows were grouped together. The taxa whose relative abundance varied across estrous cycle phases are presented in Fig 4.

**Fig 4:**
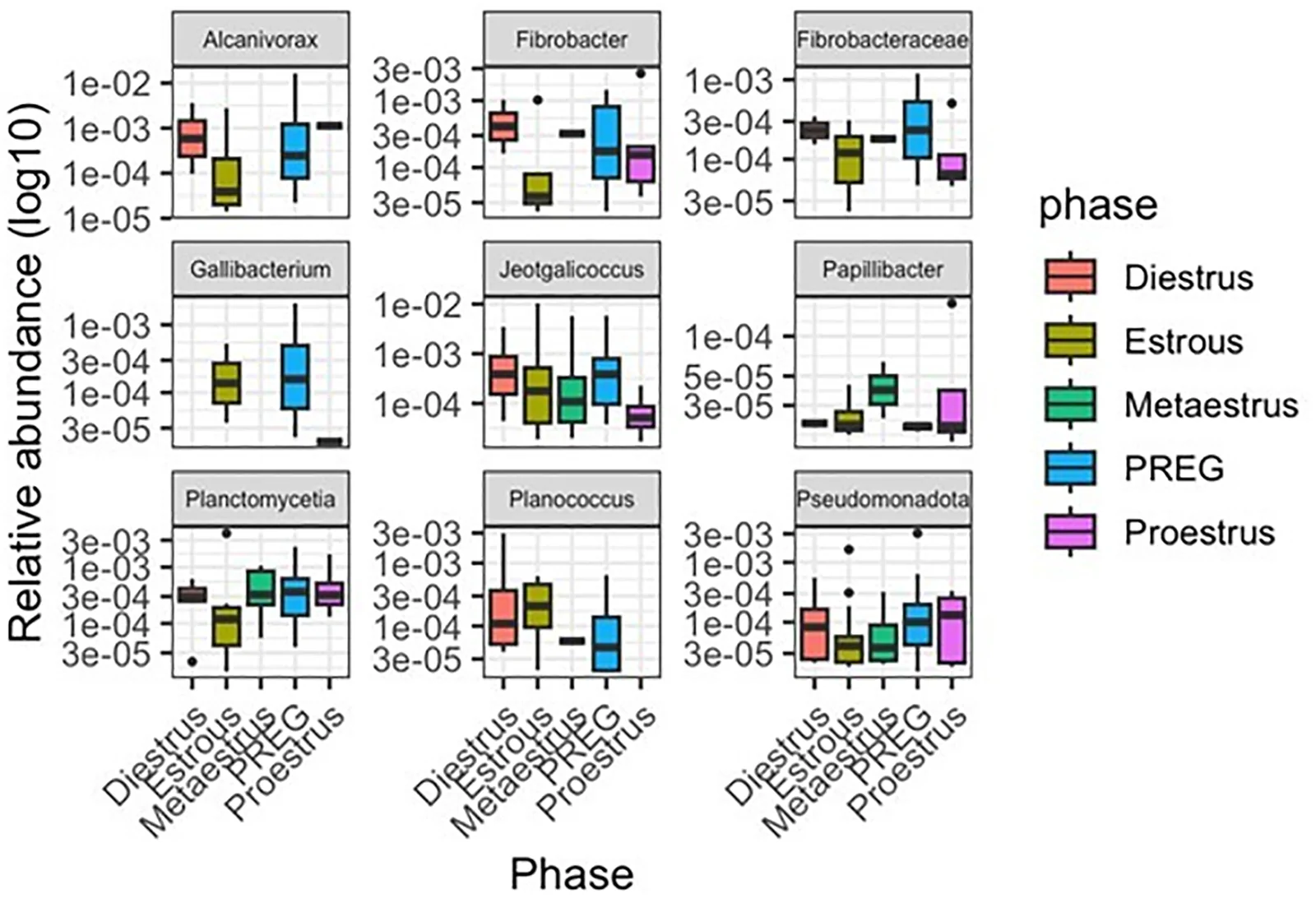
Differentially abundant taxa across reproductive stages and pregnancy status. Relative abundances of bacterial taxa showing significant differences among reproductive stages according to LinDA analysis. D0 = estrus and artificial insemination; D3 = metestrus; D15 = diestrus; D19 = proestrus (strictly within the non-pregnant cohort); PREG = samples collected from cows subsequently diagnosed as pregnant at D31. Boxes represent the interquartile range, horizontal lines indicate the median, and whiskers represent data dispersion**.**

### Impact of the estrous cycle on the alpha diversity of the vaginal microbiota

Alpha-diversity analysis showed that the number of operational taxonomic units (OTUs) did not differ significantly between the estrous cycle phases and gestation (Kruskal–Wallis, p = 0.2665; Fig 5A). Despite the absence of statistically significant differences, a trend toward greater richness was observed during estrus. Shannon diversity did not reach significance (Kruskal–Wallis, p = 0.222) (Fig 5B). These patterns suggest that although natural hormonal fluctuations throughout the cycle modestly influence microbial diversity, individual heterogeneity outweighs the effects attributable to physiological phases.

**Fig 5:**
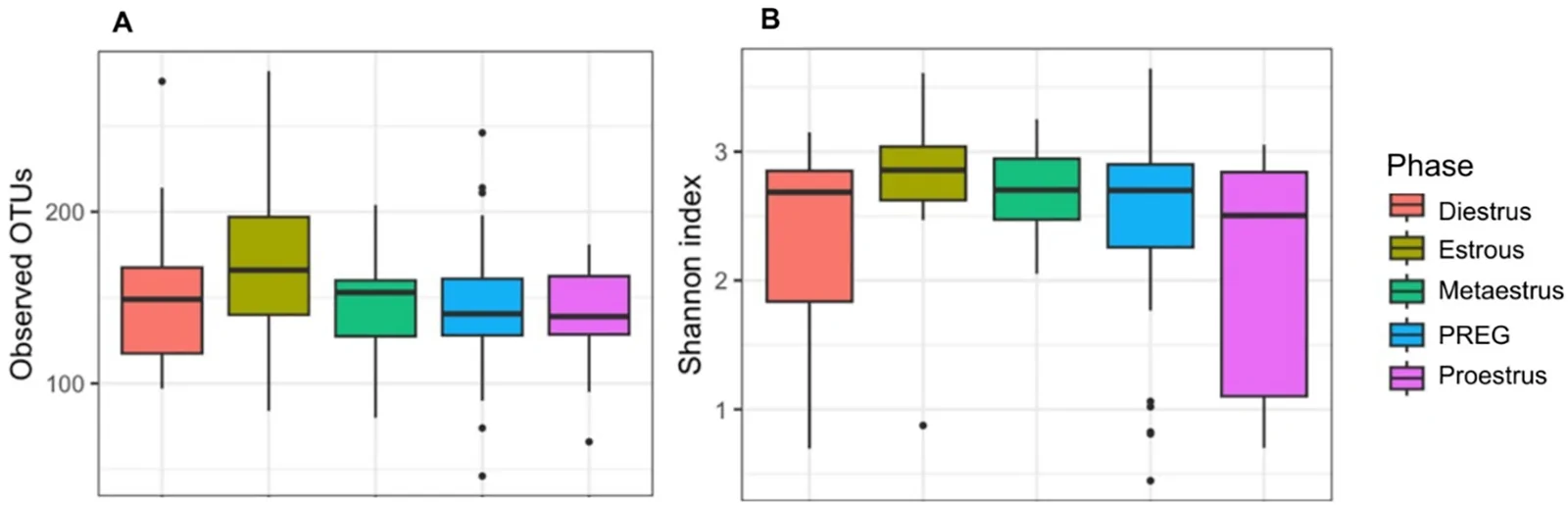
Alpha diversity of the vaginal microbiota across reproductive stages and pregnancy status. (A) Number of observed operational taxonomic units (OTUs) and (B) Shannon diversity index in vaginal samples collected throughout the reproductive cycle. D0 = estrus and artificial insemination; D3 = metestrus; D15 = diestrus; D19 = proestrus (with proestrus evaluated strictly in non-pregnant cows); PREG = samples collected from cows subsequently diagnosed as pregnant at D31. Boxes represent the interquartile range, horizontal lines indicate the median, and whiskers represent data dispersion.

### Community structure across reproductive cycle

The beta-diversity analysis based on Bray–Curtis distance identified significant differences in microbial community structure across specific phases. Specifically, comparisons between Estrus and Proestrus (evaluated exclusively within the non-pregnant cohort; p = 0.02997, PERMANOVA) and between Estrus and Diestrus (p = 0.02398, PERMANOVA) revealed divergences in bacterial profiles (Fig 6). Despite the statistical difference, there was no clear separation between the groups along the main axes of the PCoA, with considerable dispersion within each stage, suggesting that other factors, such as individual variation, may influence vaginal microbiota composition.

**Fig 6:**
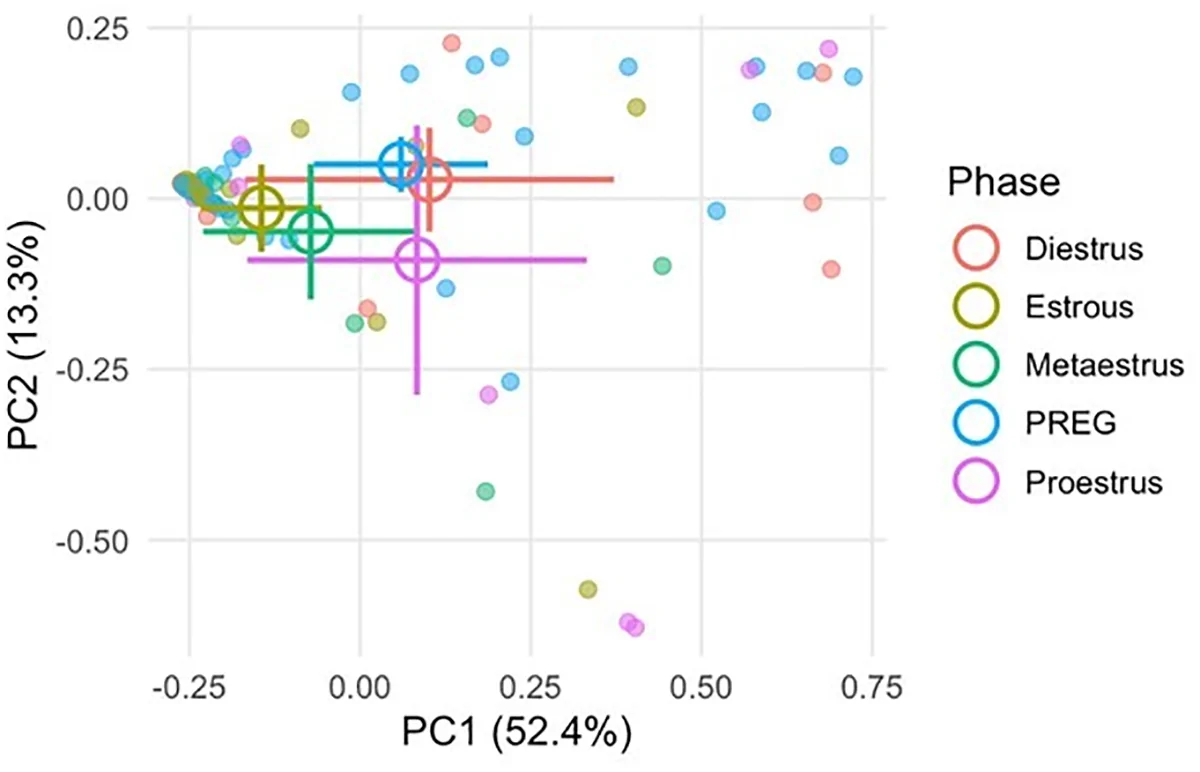
Principal Coordinate Analysis (PCoA) of the vaginal microbiota across reproductive stages and pregnancy status. Principal Coordinate Analysis based on Bray–Curtis dissimilarity illustrating differences in microbial community composition among reproductive stages. D0 = estrus and artificial insemination; D3 = metestrus; D15 = diestrus; D19 = proestrus [non-pregnant cohort only]; PREG = samples collected from cows subsequently diagnosed as pregnant at D31. Each point represents an individual sample, and ellipses indicate the 95% confidence interval around group centroids.

### Identification of potential microbial biomarkers associated with cycle stages and pregnancy

In the pregnant group (PREG), two taxa were significantly enhanced: Muribaculum (p = 0.003) and *Hoministercoradaptatus* (p = 0.016). Although both taxa were detected at very low relative abundances, their abundance was higher in pregnant cows than in non-pregnant cows. *Muribaculum* showed a mean relative abundance of 0.000082 ± 0.000109 in pregnant cows compared with 0.000008 ± 0.000014 in non-pregnant cows, whereas *Hoministercoradaptatus* exhibited a mean relative abundance of 0.000100 ± 0.000227 and 0.000003 ± 0.000009, respectively.

These findings suggest that these taxa may represent microbial signatures associated with gestational status, although further studies are required to validate their potential as pregnancy biomarkers.

## Discussion

This study investigated the vaginal microbiota of high-producing dairy cows throughout the physiological stages of the estrous cycle and early gestation, without external hormonal interference. This scenario is relevant because it enables characterization of natural microbial variation during the reproductive cycle, thereby avoiding potential alterations induced by synchronization protocols. In general, the results indicated that the bovine vaginal microbiota is characterized by high individual variability, whereas the effects of reproductive phases were subtle, with greater effects at the estrous phase.

Alpha-diversity analysis revealed no significant differences in richness (observed OTUs) or diversity (Shannon index) between the phases, although there was a tendency toward greater diversity during estrus. This stability of diversity throughout the cycle has already been described in some previous studies in cattle [15] and in mares [16]. The present result suggests that, in cattle, physiological hormonal fluctuations may not exert a sufficiently intense impact to robustly remodel the microbial structure. However, our findings contrast with studies reporting variations in the microbiota across estrous cycle phases in cattle [17].

The absence of significant differences in richness and Shannon diversity suggests that physiological endocrine fluctuations do not substantially alter the overall complexity of the vaginal microbial ecosystem. Rather than promoting large-scale gains or losses of taxa, hormonal changes may selectively influence the abundance of specific bacterial groups while maintaining community richness and diversity. This interpretation is supported by the beta-diversity and differential abundance analyses, which detected compositional rearrangements despite stable alpha-diversity metrics.

Beta diversity analysis revealed significant differences between specific phases, particularly between estrus and proestrus, and between estrus and diestrus. Despite the absence of well-defined clusters in the PCoA plot, PERMANOVA results suggested that microbial composition is influenced in a discrete manner by hormonal dynamics, particularly by the rise in estradiol around estrus and the predominance of progesterone during diestrus. These findings are consistent with the physiology of the bovine reproductive tract, which is characterized by cyclic changes in mucosal permeability, vaginal secretion, immune cell influx, and endocrine signaling, all of which can selectively modulate certain bacterial groups without necessarily causing a profound reorganization of the overall community structure [2,18].

Reproductive stage explains only a small proportion of the overall variation in the vaginal microbiota. This pattern is commonly observed in microbiome studies where subtle ecological shifts occur within a highly heterogeneous microbial ecosystem [15,19]. Therefore, although hormonal fluctuations may contribute to community restructuring, individual animal characteristics appear to be the dominant drivers of microbiota composition.

In this context, it is important to note that hormonal changes, whether natural or experimentally induced, may intensify these rearrangements. Evidence shows that estradiol can influence the physicochemical properties of the vaginal mucosa and modulate specific microbial species [7], while exogenous progesterone selectively alters bacterial abundances in synchronized cows [3]. Furthermore, endogenous variations in E2 and P4 have been associated with relevant uterine microbial changes [8]. Therefore, these hormones affect mucus characteristics, immune response, and local physicochemical conditions, potentially favoring specific microbial profiles [1,2,4,19–21]. Such evidence indicates that studies conducted under artificial hormonal manipulation may overestimate the magnitude of microbial changes relative to the physiological cycle, underscoring the value of our results obtained from untreated animals.

The findings from the present study are consistent with previous multivariate analyses reporting microbial differences across reproductive cycle phases. For instance, Ault et al., (2019) reported compositional differences in the bovine uterine microbiota between follicular and luteal stages based on beta diversity metrics. Similarly, Quereda et al., (2020) described hormone-associated shifts in the vaginal microbiota of ewes across the estrous cycle, particularly between follicular and luteal phases.

Despite these similarities, important methodological differences should be considered. Several previous studies evaluated only two broad hormonal states (follicular vs. luteal) or used hormonally synchronized animals, conditions that may accentuate microbial shifts. In contrast, the present study evaluated multiple physiological stages of the bovine reproductive cycle in untreated animals, allowing the detection of more discrete microbial differences between adjacent phases, particularly between estrus and proestrus (in non-pregnant cows) or diestrus. These findings suggest that under natural endocrine conditions the restructuring of the reproductive tract microbiota may be relatively subtle.

One limitation of the present study is that sampling was performed at predefined time points following artificial insemination rather than at hormonally confirmed reproductive stages for each individual animal. Consequently, Day 19 corresponded to a true proestrus stage only in non-pregnant cows undergoing luteolysis, whereas cows subsequently diagnosed as pregnant remained under a progesterone-dominated endocrine environment. To account for this biological distinction, samples from pregnant cows were analyzed separately from the estrous cycle phase categories. Additional sampling immediately preceding the subsequent estrus could provide a more detailed characterization of follicular-phase microbiota dynamics and should be considered in future studies.

Discrepancies between studies may reflect methodological variation, including the 16S gene region sequenced, breed differences, small variations in collection timing, and differences in sequencing depth. Furthermore, the wide variation observed within each reproductive group suggests that individual factors, such as reproductive history, local immunocompetence, basal microbiota composition, and anatomical characteristics, likely exert greater influence on the vaginal community than the estrous cycle phase itself [5]. These contrasts reinforce the need for greater methodological standardization and in-depth characterization of the bovine reproductive microbiota.

Beyond its role in reproductive health, the vaginal microbiota has also been hypothesized to participate in semiochemical communication in cattle. A recent review proposed that cervico-vaginal microorganisms may contribute to the production or modification of volatile compounds involved in pheromone signaling and reproductive behavior [22]. Although direct evidence in cattle remains limited, experimental studies in ewes [23], bitches [24], and rats [25] have demonstrated that disruption of the normal vaginal microbiota by antibiotic treatment reduces female attractiveness to males. Therefore, the relatively subtle microbial fluctuations observed in the present study may be consistent with the maintenance of microbial functions associated with reproductive communication under natural physiological conditions.

Differential analysis identified only a couple of taxa associated with cycle phases and gestation, reinforcing the conclusion that microbial changes are subtle and restricted to specific groups. The increased abundance of *Muribaculum* and *Hoministercoradaptatus* in pregnant cows suggests that early gestation can alter the vaginal microbiota. Interestingly, neither *Muribaculum* nor *Hoministercoradaptatus* have been consistently reported among the predominant members of the bovine reproductive tract microbiota in previous studies. Most investigations of the vaginal microbiota in cattle have identified genera belonging to Firmicutes, Bacteroidetes, and Proteobacteria as the dominant taxa, whereas these genera have rarely been described in the reproductive tract environment [9–11,15,19].

*Muribaculum* belongs to the family Muribaculaceae, a bacterial group commonly found in the intestinal microbiota of mammals and frequently associated with carbohydrate metabolism and host–microbiome interactions [26,27]. Although members of this family have been extensively described in gut microbial communities, their occurrence and potential role in the bovine reproductive tract remain largely unknown. Likewise, information regarding *Hoministercoradaptatus* is scarce, and to our knowledge there are currently no reports describing a consistent association of this taxon with the bovine reproductive tract microbiota.

Therefore, the enrichment of these taxa in pregnant animals observed in the present study may represent a previously unrecognized microbial signature associated with early pregnancy. However, given the limited information currently available regarding their ecological role in the bovine reproductive tract, further studies are required to confirm these findings and determine whether these microorganisms may serve as reliable biomarkers of pregnancy establishment.

Previous studies indicate that pregnant cows tend to have less diverse and more stable microbiotas, with a reduction in inflammatory taxa such as *Histophilus* and *Fusobacterium*, and an enrichment of groups associated with a more tolerant immune environment, such as Rikenellaceae and some strains of Muribaculaceae [6,8,9,19].

In addition, the literature indicates that certain bacterial communities can serve as biomarkers of gestation in cattle, thereby complementing the interpretation of our results. More stable microbial profiles with lower abundance of opportunistic pathogens have been associated with higher conception rates, while subtle changes in groups such as *Ureaplasma* and some fermenting families (e.g., Ruminococcaceae) have been linked to better conditions for artificial insemination, especially when related to favorable hormone levels during estrus [7]. Taken together, these studies reinforce the idea that the presence of specific taxa during gestation or at critical moments of fertility may reflect physiological adjustments specific to reproduction, corroborating the notion that microbial biomarkers, although subtle, carry functional relevance and can distinguish physiological phases with greater precision than isolated diversity metrics [9,28].

Taken together, the findings of the present study support the hypothesis that the bovine vaginal microbiota is highly variable between cows and has a relatively stable core throughout the estrous cycle, with subtle variations reflecting natural physiological adjustments. Furthermore, the high individual variability reinforces the need for broad sampling and experimental designs that control for factors such as age, genetic background, breed effects, productive stage, reproductive history, and environmental conditions, all of which can influence the microbiota [5,28].

From an applied perspective, understanding these nuances is essential for optimizing reproductive strategies. If, on the one hand, the relative stability of the microbiota throughout the cycle suggests that hormonal interventions may exert more intense effects than the physiological cycle, on the other hand, the identification of potential gestational markers and microvariations associated with estrus opens up space for the development of microbial indicators of gestation and the suitability of the vaginal environment for procedures such as artificial insemination. The characterization of a microbial profile favorable to conception, or, conversely, of profiles associated with reproductive failures, may in the future support monitoring and precision intervention practices in bovine reproduction.

Finally, the absence of marked differences between phases and the clear predominance of individual effects underscore the importance of investigating the determinants of the “ideal” or functional microbial composition of the reproductive tract. Longitudinal studies that track microbial changes before, during, and after conception, as well as integrated approaches combining metagenomics, metabolomics, and physiological parameters, can substantially deepen understanding of the microbiota-fertility interaction. The presence of several genera (e.g., *Fibrobacter*) that are not consistently reported in this niche was unexpected, and further studies are necessary to determine whether it is of fecal origin. This knowledge will be important not only to advance the reproductive management of high-producing dairy cows, but might also have implications for the intestinal colonization of calves.

It is important to mention that the methods used for microbiota analysis in the current study (amplicom sequencing) have limitations. The sequencing of a short region of the 16S rRNA gene has limited capacity to classify bacteria to the species level. Although bioinformatics is an evolving field and the most recent studies have employed more sensitive parameters (e.g., ASVs and 99% OTU clustering), we opted for a standard approach (i.e., 97% clustering) as a trade-off to increase comparability with the vast majority of studies investigating the bovine vaginal environment. The different approaches should not influence the conclusions of this study, which focuses on the biological interpretation driving microbiota differences.

In addition, the analysis of relative abundances may not reflect the true variation in each taxon in the environment. For example, the fluctuations observed in *Histophilus* spp. might have been caused by increases or decreases in the proportion of other taxa, which culminated in a change in their relative abundance. Thus, the use of quantitative methods is necessary to confirm those findings and to further explore the capacity of the markers reported in the study to discriminate the different phases. Finally, while using a single farm is desirable to avoid introducing additional variables into the results, larger studies are necessary before the current findings can be extrapolated.

## Conclusion

This study demonstrated that the vaginal microbiota of high-producing dairy cows, evaluated under physiological conditions and without hormonal interference, exhibits high individual variability and only discrete fluctuations associated with the estrous cycle phases. These results contribute to a better understanding of microbial dynamics within the bovine reproductive tract and provide additional insight into microbial signatures associated with reproductive status and early pregnancy establishment under natural endocrine conditions.

## Supporting information

S1 File Inclusivity in global research questionnaire. (DOCX)

S2 File Signed Statement of Acknowledgment and Authorization from the owner of the farm and animals. (PDF)

S3 File OTU abundance table showing the number of reads of each operational taxonomic unit (OTU) across samples. (TXT)

S4 File Taxonomy file providing the taxonomic classification assigned to each OTU, from phylum to genus. (TXT)

S5 File Metadata file containing characteristics associated with each sample. (TXT)

## References

[1] Adnane M, Chapwanya A. A review of the diversity of the genital tract microbiome and implications for fertility of cattle. Animals. 2022. doi: 10.3390/ani12040460 PMC8868056 35203168

[2] Quereda JJ, Barba M, Mocé ML, Gomis J, Jiménez-Trigos E, García-Muñoz Á. Vaginal microbiota changes during estrous cycle in dairy heifers. Front Vet Sci. 2020. doi: 10.3389/fvets.2020.00371 PMC7350931 32719814

[3] Quadros DL, Zanella R, Bondan C, Zanella GC, Facioli FL, da Silva AN, et al. Study of vaginal microbiota of Holstein cows submitted to an estrus synchronization protocol with the use of intravaginal progesterone device. Res Vet Sci. 2020;131:1–6. doi: 10.1016/j.rvsc.2020.03.027 32278134

[4] Zhaoxin T, Moreno Muñoz A, Ordesa L, Huang M, Tan tanzhjin Z, Yu R. Sex hormones influence the intestinal microbiota composition in mice. 10.3389/fmicb.2022.964847 PMC9659915 36386696

[5] Dias NW, Poole R, Soffa DR, Brown KJH. Dynamic principles of the microbiome and the bovine vagina: A review. Frontiers in Microbiology. 2024. doi: 10.3389/fmicb.2024.1434498 PMC11655496 39703711

[6] Wang N, Chen L, Yi K, Zhang B, Li C, Zhou X. The effects of microbiota on reproductive health: A review. Crit Rev Food Sci Nutr. 2024;64(6):1486–507. doi: 10.1080/10408398.2022.2117784 36066460

[7] Messman RD, Contreras-Correa ZE, Paz HA, Perry G, Lemley CO. Vaginal bacterial community composition and concentrations of estradiol at the time of artificial insemination in Brangus heifers. J Anim Sci. 2020;98(6):skaa178. doi: 10.1093/jas/skaa178 32515480 PMC7281872

[8] Poole RK, Pickett AT, Oliveira Filho RV, de Melo GD, Palanisamy V, Chitlapilly Dass S, et al. Shifts in uterine bacterial communities associated with endogenous progesterone and 17β-estradiol concentrations in beef cattle. Domest Anim Endocrinol. 2023;82:106766. doi: 10.1016/j.domaniend.2022.106766 36182815

[9] Webb EM, Holman DB, Schmidt KN, Pun B, Sedivec KK, Hurlbert JL, et al. Sequencing and culture-based characterization of the vaginal and uterine microbiota in beef cattle that became pregnant or remained open following artificial insemination. Microbiol Spectr. 2023;11(6):e0273223. doi: 10.1128/spectrum.02732-23 37921486 PMC10714821

[10] Adnane M, Chapwanya A. Microbial gatekeepers of fertility in the female reproductive microbiome of cattle. Int J Mol Sci. 2024;25(20):10923. doi: 10.3390/ijms252010923 39456706 PMC11507627

[11] Chen S-Y, Deng F, Zhang M, Jia X, Lai S-J. Characterization of vaginal microbiota associated with pregnancy outcomes of artificial insemination in dairy cows. J Microbiol Biotechnol. 2020;30(6):804–10. doi: 10.4014/jmb.2002.02010 32238772 PMC9728155

[12] Schloss PD, Westcott SL, Ryabin T, Hall JR, Hartmann M, Hollister EB, et al. Introducing mothur: open-source, platform-independent, community-supported software for describing and comparing microbial communities. Appl Environ Microbiol. 2009;75(23):7537–41. doi: 10.1128/AEM.01541-09 19801464 PMC2786419

[13] Kozich JJ, Westcott SL, Baxter NT, Highlander SK, Schloss PD. Development of a dual-index sequencing strategy and curation pipeline for analyzing amplicon sequence data on the MiSeq Illumina sequencing platform. Appl Environ Microbiol. 2013;79(17):5112–20. doi: 10.1128/AEM.01043-13 23793624 PMC3753973

[14] Zhou H, He K, Chen J, Zhang X. LinDA: Linear models for differential abundance analysis of microbiome compositional data. Genome Biol. 2022;23(1):95. doi: 10.1186/s13059-022-02655-5 35421994 PMC9012043

[15] Laguardia-Nascimento M, Branco KMGR, Gasparini MR, Giannattasio-Ferraz S, Leite LR, Araujo FMG, et al. Vaginal microbiome characterization of nellore cattle using metagenomic analysis. PLoS One. 2015;10(11):e0143294. doi: 10.1371/journal.pone.0143294 26599789 PMC4657983

[16] Barba M, Martínez-Boví R, Quereda JJ, Mocé ML, Plaza-Dávila M, Jiménez-Trigos E, et al. Vaginal microbiota is stable throughout the estrous cycle in arabian maress. Animals (Basel). 2020;10(11):2020. doi: 10.3390/ani10112020 33153053 PMC7692283

[17] Ault TB, Clemmons BA, Reese ST, Dantas FG, Franco GA, Smith TPL, et al. Uterine and vaginal bacterial community diversity prior to artificial insemination between pregnant and nonpregnant postpartum cows1. J Anim Sci. 2019;97(10):4298–304. doi: 10.1093/jas/skz210 31250893 PMC6776308

[18] Srinivasan M, Adnane M, Archunan G. Significance of cervico-vaginal microbes in bovine reproduction and pheromone production – A hypothetical review. Res Vet Sci. 2021;:66–71. doi: 10.1016/j.rvsc.2021.01.003 33450498

[19] Deng F, McClure M, Rorie R, Wang X, Chai J, Wei X, et al. The vaginal and fecal microbiomes are related to pregnancy status in beef heifers. J Anim Sci Biotechnol. 2019;10:92. doi: 10.1186/s40104-019-0401-2 31857897 PMC6909518

[20] Dong M, Dong Y, Bai J, Li H, Ma X, Li B. Interactions between microbiota and cervical epithelial, immune, and mucus barrier. Frontiers Cell Infection Microbiol. 2023. doi: 10.3389/fcimb.2023.1124591 PMC9998931 36909729

[21] Izadifar Z, Cotton J, Chen S, Horvath V, Stejskalova A, Gulati A, et al. Mucus production, host-microbiome interactions, hormone sensitivity, and innate immune responses modeled in human cervix chips. Nat Commun. 2024;15(1):4578. doi: 10.1038/s41467-024-48910-0 38811586 PMC11137093

[22] Srinivasan M, Adnane M, Archunan G. Significance of cervico-vaginal microbes in bovine reproduction and pheromone production - A hypothetical review. Res Vet Sci. 2021;135:66–71. doi: 10.1016/j.rvsc.2021.01.003 33450498

[23] Ungerfeld R, Silva L. The presence of normal vaginal flora is necessary for normal sexual attractiveness of estrous ewes. Appl Anim Behav Sci. 2005;93:245–50. doi: 10.1016/J.APPLANIM.2004.11.014

[24] Dziecioł M, Nizański W, Stańczyk E, Kozdrowski R, Najder-Kozdrowska L, Twardoń J. The influence of antibiotic treatment of bitches in oestrus on their attractiveness to males during mating. Pol J Vet Sci. 2013;16(3):509–16. doi: 10.2478/pjvs-2013-0071 24195286

[25] Merkx J, Slob AK, van der Werff ten Bosch JJ. Vaginal bacterial flora partially determines sexual attractivity of female rats. Physiol Behav. 1988;44(1):147–9. doi: 10.1016/0031-9384(88)90359-9 3237810

[26] Lagkouvardos I, Lesker TR, Hitch TCA, Gálvez EJC, Smit N, Neuhaus K, et al. Sequence and cultivation study of Muribaculaceae reveals novel species, host preference, and functional potential of this yet undescribed family. Microbiome. 2019;7(1):28. doi: 10.1186/s40168-019-0637-2 30782206 PMC6381624

[27] Zhu Y, Chen B, Zhang X, Akbar MT, Wu T, Zhang Y. Exploration of the Muribaculaceae family in the gut microbiota: Diversity, metabolism, and function. Nutrients. 2024. doi: 10.3390/nu16162660 PMC11356848 39203797

[28] Fragomeni B, Hird SM, Zezeski AL, Geary TW, McCoski SR, Hay EH. Vaginal microbiome is associated with breed and pregnancy status in beef cattle. Animals (Basel). 2026;16(6):874. doi: 10.3390/ani16060874 41897851 PMC13023300

