# Supplementary material for: The impact of the estrous cycle on the vaginal microbiota of dairy cows
Source: PLoS One. 2026 Oct 5;21(10):e0359551. doi: 10.1371/journal.pone.0359551 (PMC13637902; doi:10.1371/journal.pone.0359551)
Supplement: Supplementary file 1 [file pone.0359551.s002.pdf]

Supporting Information

The document below provides additional details regarding owner consent and the experimental procedures performed

The document provided as Supporting Information consists of the *Statement of Acknowledgment and Authorization* signed by the owner of the farm and the animals used in the study described in the present manuscript. This document was obtained prior to the initiation of the research activities and formally authorized the inclusion of the animals in the project.

The signed statement describes the objectives of the research project and provides detailed information regarding the procedures to be performed, as well as their potential consequences and implications for the animals involved. The document also confirms that the owner was adequately informed about the study protocol and voluntarily agreed to the participation of the animals in the research.

TERMO DE CIÊNCIA E AUTORIZAÇÃO

Eu Gustavo Martins Gomes dos Santos, RG: 87737543, responsável pelos bovinos (espécie animal) da propriedade rural Fazenda Santa Maria, autorizo a participação dos animais no projeto de pesquisa intitulado (a) **CARACTERIZAÇÃO DO MICROBIOMA REPRODUTIVO E O IMPACTO NA FERTILIDADE BOVINA** sob coordenação do pesquisador da Universidade Estadual de Londrina que assina abaixo. Fui esclarecido sobre os objetivos do projeto, que são:

- Avaliar aspectos reprodutivos que possam alterar a microbiota de bovinos leiteiros utilizando o sequenciamento de nova geração.
- Investigar a influência da fase do ciclo estral sobre a composição bacteriana vaginal de fêmeas bovinas.
- Estabelecer associações entre comunidades bacterianas específicas e a infertilidade de fêmeas bovinas.
- Associar a população bacteriana ao desempenho reprodutivo, comparando a microbiota vaginal de vacas prenhes e vacas que não conseguiram emprenhar, diagnosticadas vazias após a Inseminação Artificial.

Também fui esclarecido quanto aos procedimentos a serem empregados e suas possíveis conseqüências sobre o animal, os quais serão:

- Realização de exame de ultrassonografia transretal das vacas periodicamente, a fim de identificar as fases do ciclo estral, totalizando cerca de 60 dias de acompanhamento por animal.
- Coleta de amostras do conteúdo vaginal por meio de uma cotonete estéril, indolor para o animal.
- Inseminação artificial sem a utilização hormonal, não apresentando risco para o animal.

Tenho conhecimento de que os resultados obtidos serão divulgados exclusiva e anonimamente em publicações científicas; e de que tenho o direito e a liberdade de suspender essa autorização a qualquer momento que julgue necessário.

Londrina, 10 de Outubro de 2024.

Assinatura do responsável pelos animais

Prof. Dr. Marcelo Marcondes Seneda
Coordenador do projeto e orientador
CCA/DCV
Universidade Estadual de Londrina - UEL
Telefone: (43) 33715622

Below is the English translation of the document above

STATEMENT OF AWARENESS AND AUTHORIZATION

I, Gustavo Martins Gomes dos Santos, ID No. (RG): 87737543, responsible for the cows (animal species) from the rural property Fazenda Santa Maria, hereby authorize the participation of the animals in the research project entitled **"CHARACTERIZATION OF THE VAGINAL MICROBIOTA OF DAIRY COWS AT DIFFERENT STAGES OF THE ESTROUS CYCLE,"** under the coordination of the researcher from the State University of Londrina who signs below.

I have been informed about the objectives of the project, which are:

• To evaluate reproductive aspects that may alter the microbiota of dairy cattle using next-generation sequencing.
• To investigate the influence of the stage of the estrous cycle on the vaginal bacterial composition of bovine females.
• To establish associations between specific bacterial communities and infertility in bovine females.
• To associate the bacterial population with reproductive performance by comparing the vaginal microbiota of pregnant cows and cows that failed to become pregnant, diagnosed as non-pregnant after Artificial Insemination.

I have also been informed about the procedures to be performed and their possible consequences for the animal, which will include:

• Periodic transrectal ultrasonographic examination of the cows to identify the stages of the estrous cycle, totaling approximately 60 days of monitoring per animal.
• Collection of vaginal tissue samples using a sterile swab, which is painless for the animal.
• Artificial insemination without the use of hormones, presenting no risk to the animal.

I acknowledge that the results obtained will be disclosed exclusively and anonymously in scientific publications, and that I have the right and freedom to withdraw this authorization at any time I deem necessary.

Londrina, 10 of October of 2024.

Gustavo Martins Gomes dos Santos

The person responsible for animals

Prof. Dr. Marcelo Marcondes Seneda
Project Coordinator and Advisor
CCA/DCV
State University of Londrina – UEL
Telephone: (43) 3371-5622
